# Less Talk More Chalk: A Retrospective Review of Rock Climbing-Related Trauma

**DOI:** 10.7759/cureus.60033

**Published:** 2024-05-10

**Authors:** Hanien Samara, Andrew McCague, Austin Henken-Siefken

**Affiliations:** 1 General Surgery and Trauma Surgery, Western University of Health Sciences, Pomona, USA; 2 Trauma and Acute Care Surgery, Desert Regional Medical Center, Palm Springs, USA; 3 Surgery, Desert Regional Medical Center, Palm Springs, USA

**Keywords:** trauma center, lower extremity injury, trauma fracture, rock climbing, falls from heights

## Abstract

Introduction

Climbing is a strength and strategy-driven sport that has greatly increased in popularity over the last decade, partially due to its debut in the Tokyo 2020 Summer Olympics. With an increasing number of new climbers and the emergence of recreational indoor climbing facilities, fall injury risk remains a legitimate concern within the climbing community. This study evaluates the pattern of injury in trauma patients presenting to the Desert Regional Medical Center, a level 1 trauma center in Palm Springs, CA, following falls from height while rock climbing.

Methods

Our study retrospectively investigated a de-identified dataset on trauma patients at the Desert Regional Medical Center, a level 1 trauma center located in Palm Springs, CA, from 2016 to 2021. This analysis focused on 75 patients who presented following falls from height while rock climbing. We reviewed several parameters, including patient demographics, Injury Severity Score (ISS), hospital length of stay (LOS), injury type, and patient outcomes. Descriptive statistics including median values, standard deviations (SD), and P-values were assessed via Microsoft Excel. Several paired, one-tailed t-tests and a Pearson's correlation test were also conducted to further evaluate the association between variables within the dataset.

Results

In this retrospective analysis of patients presenting to the emergency department post-fall from heights while rock climbing, the patient profile was predominantly younger or middle-aged climbers under 60 years old (65, 86.7%). The mean patient age was 37 years old. The majority of patients were non-Hispanic (69, 92%), noting a male predominance (57, 76%). Most patients (60, 80%) required partial trauma code status. Hospitalization was required for most individuals (67, 89.3%), with several requiring intensive care unit (ICU) admission (29, 38.7%). The average hospital LOS was 6.7 amongst all admissions. Patients requiring LOS greater than 10 days had a higher average ISS (12.9) when compared to climbers with shorter lengths of admission (ISS of 10.4). There was no significant difference in ISS between younger patients (ISS of 9.3) and those 60 and older (ISS of 10.6). The most common critical injury was lower extremity fracture (36, 48%), noting no significant increase in injury incidence over the last five years.

Conclusion

Rock climbers who experience falls from cliffs are most at risk for a lower extremity fracture. Demographically, a majority of injured climbers in this study were young males, who may exhibit risk-taking behavior. To better prevent critical injuries within the climbing sector, we encourage an increase in safety measures (crash mats, harnessing) and the implementation of a new climber education program.

## Introduction

Rock climbing, a strength and resistance training sport, has gained increasing popularity over the last decade and was added to the Olympic Games in 2020 [1,2]. Consisting of various subgroups, climbing may be categorized by indoor/outdoor classification and climbing type. Climbing type focuses on the heights scaled and protection equipment used. The two main genres include bouldering and free climbing. Bouldering includes scaling heights of 10-15 feet with crash pads placed below the route, whereas free climbing details scaling heights of 20-40 feet while harnessed into belaying devices with a partner holding the opposite rope end [3]. With an increasing number of climbers and the establishment of more difficult routes, concerns regarding injury from falls remain prominent within the climbing community. Approximately 3,816 rock climbing-associated injuries are seen in US emergency departments annually [4]. Traditionally, injuries resulting from falls from heights are placed within two distinct categories: direct impact and deceleration-type injuries. Direct impact injuries often consist of fractures, whereas deceleration-type injuries lead to internal damage [5]. The purpose of this study is to analyze injury patterns of trauma patients presenting to the Desert Regional Medical Center, a level 1 trauma center, following falls from height while rock climbing. We hope that in doing so, we can gain a greater understanding of the risk rock climbing entails and better determine the injury pattern associated with recreational climbing.

## Materials and methods

As a level 1 trauma center within close proximity to several national parks and rock climbing destinations, the Desert Regional Medical Center is an ideal site to survey our population of interest (rock climbers). Initially, over 3,000 patients presented to the Desert Regional Medical Center emergency department based on county triage policies following falls from height. Per hospital policy, all patients were registered into the trauma registry with incidents ranging from January 2016 to December 2021. Each of the patients from the registry was then entered into a de-identified dataset, and the authors surveyed/selected only patients (climbers) who suffered falls while rock climbing to participate in this retrospective study. Of the 3,000 initial patients, 75 met the inclusion criteria and were included in this study. The de-identified dataset included patient age, sex, disposition, Injury Severity Score (ISS), mode of transportation to the emergency department, injury type, hospital length of stay (LOS), length of intensive care unit (ICU) stay, ventilator days, need for blood transfusions, need for surgery, and initial workup (labs and imaging). No patient identifiers were available to the investigators, and patients were not contacted any time throughout this study. Data was secured and protected in password-protected files. For this study, we focused on patient demographics, trauma code activation, injury classification, and admission status. Descriptive statistics including mean values, standard deviations (SD), and P-values were quantified via Microsoft Excel. Researchers ran several paired, one-tailed t-tests to analyze the possible association between several values listed in the dataset. Additionally, a Pearson's correlation test was conducted to determine the possible association between injury occurrence and year.

## Results

Seventy-five patients met the study criteria and were included within the analysis. All patients presented with signs of life upon emergency department arrival. Demographically, a majority of patients (65, 86.7%) were under the age of 60, with a mean patient age of 37 years old. With reference to patient sex, 57 (76%) patients identified as male, 17 (22.6%) identified as female, and one (1.3%) was unspecified. Based on race, 69 (92%) patients identified as non-Hispanic or Latino. Following triage, 10 (13.3%) climbers warranted full trauma code activation, 60 (80%) patients required a partial code, and five (6.7%) patients did not necessitate code activation. Trauma code status is set regionally and based upon physiological data. A full code is called for all patients meeting the objective major trauma criteria, whereas a partial code is initiated for all injured patients not meeting full trauma activation guidelines [6] (Table 1).

**Table 1:** Demographics data

Demographics	Number of patients, n	Percentage cases, %
Number of patients	75	100
Gender		
Male	57	76
Female	17	22.6
Unspecified	1	1.3
Age 60	10	13.3
Race		
Hispanic or Latino	5	6.7
Non-Hispanic or Latino	69	92
Unspecified	1	1.3
Trauma code status		
Full code	10	13.3
Partial code	60	80
No code	5	6.7

Following triage, nine (12%) patients required immediate surgical intervention and were sent to the operating room. A total of sixty-seven (89.3%) patients were admitted, 29 (38.7%) of which to the ICU. The average LOS for all admitted patients was 6.7 days, with an average LOS of 3.97 days in the ICU. Only three (4%) patients required discharge to an acute rehabilitation unit (ARU) (Table 2). Individuals with an LOS greater than 10 days had a higher average ISS (12.9) when compared to climbers with shorter lengths of admission (ISS of 10.4) (Table 3). In terms of age, there was no significant difference between ISS for patients aged 60 and/or older (ISS of 10.6) versus younger patients (ISS of 9.3) (Table 4).

**Table 2:** Admissions data ICU: intensive care unit; LOS: length of stay; SD: standard deviation; ARU: acute rehabilitation unit

Disposition	Number of patients, n	Percentage cases (75), %
Admissions	67	89.3
ICU admissions	29	38.7
Surgery	9	12
ARU (after discharge)	3	4
Death	0	0
	LOS (days)	SD (days)
Average LOS of admitted patients	6.7	6.3
Average LOS in the ICU	3.97	5.1

**Table 3:** Average ISS by LOS SD: standard deviation; ISS: Injury Severity Score; LOS: length of stay

	Average ISS	SD (ISS)	P
ISS (all patients)	9.54	6	0.05
ISS with LOS ≤10 days	9.8	5.8	
ISS with LOS >10 days	12.8	5.56	

**Table 4:** ISS by age SD: standard deviation; ISS: Injury Severity Score

	Average age (years)	SD (years)	P
Admissions	37.6	15.9	0.206
Not admitted	32.8	15.4	
Age (years)	ISS	SD (years)	P
60	10.6	7.7	0.257
<60	9.3	5.8	

**Figure 1:**
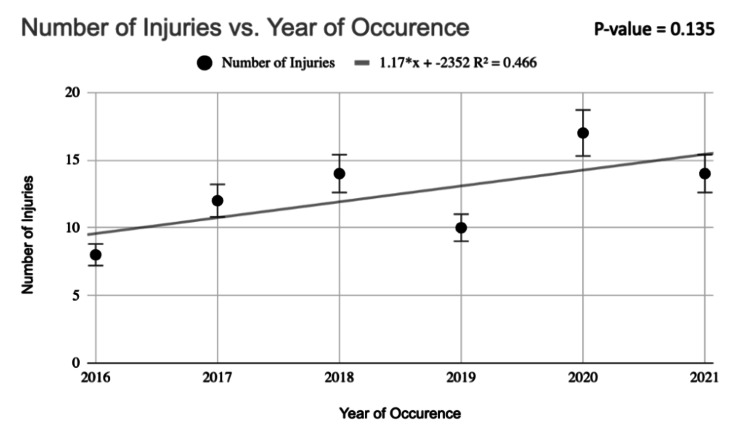
Rate of injury incidence over time

The most frequent region of injury was the extremities with 46 (61%) climbers sustaining an upper extremity and/or lower extremity wound (Table 5). The most prevalent types of injuries were lacerations, abrasions, and/or contusions with 40 (53.3%) patients. The most common critical injury was lower extremity fracture with 36 (48%) individuals displaying fractures to the foot, ankle, femur, tibia, and/or fibula. Of the total number of patients suffering from lower extremity fractures, 13 (36%) were open. Twenty-eight (37.3%) patients displayed vertebral body fractures. Twenty-two (29%) individuals sustained traumatic brain injuries (TBI) such as subdural hematomas, subarachnoid hemorrhages, and/or concussions. Thirteen (17.3%) patients were diagnosed with internal damage including but not limited to pneumo-/hemopneumothorax, organ contusion/laceration, and unspecified injury to the intra-abdominal organs. Fifteen (20%) individuals suffered skull and/or facial bone fractures. Fifteen (20%) climbers sustained rib, sternal, and/or clavicular fractures. Fourteen (18.7%) patients suffered fractures to the upper extremities (radius, ulna, humerus, scapula) and/or hand. Eleven (14.7%) individuals exhibited pelvic and/or sacral fractures, with one of those patients experiencing an open fracture. Ten (13%) patients sustained soft tissue injuries, such as joint dislocation and/or ligament/tendon injury (Table 6). When evaluating injury incidence over the last five years, we did not appreciate a significant increase or decrease in injury occurrence amongst climbers (Figure 1).

**Table 5:** Injury by the body region External injuries include superficial abrasions, lacerations, and contusions

Body region	Number of patients, n	Percentage cases (75), %
Extremity	46	61.3
External	40	53.3
Head/face	34	45.3
Back	28	37.3
Chest	15	20.0
Abdomen	10	13.3

**Table 6:** Presenting injuries on admission TBI include subdural hematomas, subarachnoid hemorrhages, and concussions. Internal injuries include pneumo-/hemopneumothorax, organ contusion/laceration, etc. TBI: traumatic brain injuries

Injury type	Number of patients, n	Percentage cases (75), %
Laceration/abrasion/contusion	40	53.3
Lower extremity fracture	36	48.0
Vertebral fracture	28	37.3
TBI	22	29.3
Skull/facial bone fracture	15	20.0
Rib/clavicular fracture	15	20.0
Upper extremity fracture	14	18.7
Internal injury	13	17.3
Pelvic/sacral fracture	11	14.7
Ligament/tendon/joint injury	10	13.3

## Discussion

Rock climbing requires both aerobic and anaerobic fitness, with the energy expenditure similar to that of moderate to intense exercise. Despite the fact that climbing has gained exceeding popularity amongst young adults, there is minimal data on youth climbers [7]. The demographics of our study support the trend of increasing youth popularity, with our data reporting higher injury rates amongst middle-aged male climbers and an average patient age of 37. Similar trends in the patient population were noted when reviewing comparable studies evaluating rock climbing injuries secondary to falls. For example, a review of outdoor climbing injuries in Sweden from 2008 to 2019 reported a mean age of 34 years old (SD 10) and a predominantly male demographic (30 male climbers out of 38 total study participants) [8]. Interestingly, such demographics are not unique to rock climbing. We have found equivalent demographics when assessing studies of falls from heights with various mechanisms of action (roofing, climbing, electrical work). Specifically, a study conducted in India surveying 861 patients presenting to the emergency department following falls from height showed a mean patient age of 36.2 and male predominance (637, 74%) [9]. We suspect injury occurrence may, in part, be due to increased risk-taking behavior in this population. A 2008 article on impulse-seeking behavior amongst rock climbers reported higher levels of risk-taking behaviors from male climbers and those with increased experience. This study included 106 athletes and measured boldness via an Impulsive-Sensation Seeking Scale. Of note, they also reported mild associations between age and impulsivity, with younger individuals exhibiting more hazardous responses [10]. 

Within this study, the most common critical injury is lower extremity fracture (36, 48%), followed by vertebral fractures (28, 37%). We suspect that patients are landing on their backs and/or attempting to land on their feet, increasing the stress placed on the vertebral bodies and the lower extremities. Whether it be due to equipment failure or climbers misjudging the situation, the reduced ability to dissipate force is likely the cause of such injuries [11]. Research conducted by orthopedic surgeon Dr. Keegan Cole, MD, further reinforces the assumption that rock climbing increases the risk of orthopedic fractures. He reports that of 975 total rock climbing injuries surveyed in his office, 366 (37.6%) were evaluated for orthopedic surgery with 157 (43%) warranting surgical intervention [12]. Although the climbing style was not included in our dataset, historically, climbers participating in top roping and/or lead climbing have the highest injury rate. For instance, a study assessing climbing gym visits in Germany from 2007 to 2011 showed 32 injuries out of 515,337 total indoor climbing sessions. Of the 32 patients, 17 (53%) were lead climbing, seven (23%) were top roping, and only six (20%) were bouldering [13]. It is believed that increasing climb height leads to a higher risk of injury. 

Following hospitalization, a minimal number of patients required relocation to an acute rehabilitation facility, with most being discharged home. Admission data did not show an increased probability of hospital admission with increased age; however, there was a correlation between higher ISS and increased hospital stay. The ISS is nationally recognized as a reliable marker of hospital LOS, reinforcing the link between the injury acuity, admission duration, and recovery time. Our ISS average of 9.54 amongst all patients is similar to that of other studies investigating outdoor rock-climbing incidents. For instance, a retrospective study at a level 1 emergency department in Switzerland reported a mean ISS of 7.5 amongst 78 climbers. Similar to our study, most injuries were fractures affecting either multiple body regions or the lower extremities. Although fewer patients within the Swiss study required hospital admission (44, 56%), hospital LOS was comparable to that of our cohort of climbers, with an average stay of seven days [14].

Although this preliminary data provides context regarding the injury patterns sustained by rock climbers, we recommend expanding this study to include a larger sample size and various geographical locations. With larger sample sizes, we may be able to extrapolate risk-taking behaviors amongst middle-aged versus older climbers and male versus female climbers. Additionally, information regarding the type of safety equipment used and the height at which the patients fell may provide better context on the mechanism of injury. Previous research investigating climbing safety equipment use found no correlation between harness type (full body or sit harness alone) and injury severity. Out of 113 injured climbers, 73 (64.6%) used a sit harness alone, whereas 40 (35.4%) used a body harness [15]. With future efforts, we hope to gain an improved perspective on the risks associated with various climbing styles and encourage appropriate safety measures for participating athletes.

## Conclusions

The goal of our retrospective study was to better understand the demographics of injured climbers and injury patterns related to recreational climbing. As indicated, a majority of injured rock climbers are middle-aged adult males, which is to be expected with the increased popularity rock climbing has had within the young adult sector. Due to the prevalence of lower extremity injury amongst the patients surveyed, we advise increasing safety measures (increased fall mat use, proper harnessing) and climber education to hopefully reduce the prevalence of critical and possibly debilitating injuries. With a majority of patients requiring hospital admission, we urge all climbers to pursue routes appropriate for their skill level.
